# Internet Use and Job Satisfaction: Evidence from China

**DOI:** 10.3390/ijerph191912157

**Published:** 2022-09-26

**Authors:** Dan Zhou, Sibo Yang, Xue Li

**Affiliations:** 1 School of Business, Xiangtan University, Xiangtan 411199, China; 2 Department of Public and International Affairs, City University of Hong Kong, Hong Kong 999077, China; 3 School of International Business and Economics, Shanghai Lixin University of Accounting and Finance, Shanghai 201620, China

**Keywords:** Internet use, urban and rural labor force, job satisfaction, time efficiency, work autonomy, China

## Abstract

We explore the causal effects of Internet use on job satisfaction using a sample of 83,012 Chinese labor force members aged 16–64 years from the China Family Panel Studies (CFPS) from 2010 to 2018. We use ordered logistic estimation and find that Internet use significantly increases job satisfaction by 3.2%. Heterogeneity analysis shows that the Internet has a more positive impact on those who are in urban areas and have higher incomes and higher education. Our results are robust after eliminating endogeneity using instrumental variables and solving the self-selection problem using the PSM method. Our mechanistic analysis leads to similar conclusions to mainstream research, where Internet use induces job satisfaction by increasing time efficiency and enhancing job autonomy. Specifically, shorter working hours boosted job satisfaction by approximately 0.3%, while working in informal places boosted job satisfaction by 5.4%. Thus, employers may consider encouraging employees to access the Internet.

## 1. Introduction

Job satisfaction refers to the feeling that work can bring psychological and physical satisfaction for employees [1]. Organizational management prioritizes enhancing job satisfaction because a high level of satisfaction attracts, recruits, motivates and retains a company’s workforce [2]. Although Internet use has become a part of most jobs, only a few scholars have estimated the direct impact of the Internet on workers’ job satisfaction and explored the mechanisms of the effects that are exerted [3].

Most existing literature on job satisfaction discusses individual satisfaction by analyzing the antecedent variables, including salary income, career prospects, education, skill levels, work organization and nature, and work relationships [4,5,6]. Few studies have explored the direct relationship, and there is a gap in China. The Chinese context provides an ideal setting for studying the relationship between Internet use and job satisfaction for several reasons.

First, China has achieved rapid economic growth, but the work time and intensity increase at the same time. The high-intensity work of laborers has brought about social problems such as sudden death and suicide from overwork, which have been fermented through the Internet and attracted widespread attention to the subjective welfare of laborers such as job satisfaction and happiness [7].

Second, the way Chinese people use the Internet is different from that of Western workers. For example, the Chinese used to communicate with colleagues by WeChat [8]. WeChat is an instant messenger, and new messages will likely be received during non-working time, affecting workers’ lives. However, most Western workers used to communicate by email. Emails can be responded to centrally based on the recipient’s wishes. The difference between the two is likely to lead to differences in job satisfaction.

Third, Chinese people are excellent targets for heterogeneity analysis because of the huge urban–rural differences in Internet use. According to the 2021 Statistical Report on Internet Development in China, 70% of Chinese residents already use the Internet, but in rural areas, only 30% have this skill [9]. This leads to a huge difference in the ability of people in different regions to access information or search for jobs through the Internet, which provides a rare opportunity to estimate the heterogeneous impact of Internet use on job satisfaction. It is important to note that the impact of the Internet on work patterns and life may have a negative effect on job satisfaction. Internet use prolongs working hours, which brings greater time efficiency [10]. In addition, excessive Internet use will also reduce people’s real-life information interaction, thereby reducing work enthusiasm [11]. Finally, the Internet is not yet the main work tool for migrant workers who are characterized by manual labor and repetitive tasks, so Internet use have little impact on job satisfaction [12].

Seashore and Tobor believe that job satisfaction is determined by a combination of external environmental factors and personal factors. The former includes the work environment and workplace relationships, while the latter includes family, education, income, age, and other personal characteristics [13]. Specifically, workers who are highly educated or have high incomes tend to have high job satisfaction [14,15]. Research findings on age are not uniform, for example, Peng et al. concluded that the effect of the Internet on older adults is not significant and negative emotions are mainly generated by younger groups [16]. However, more scholars believe that there is an inverted U-shaped relationship between age and job satisfaction because younger people are more likely to use the Internet for work and older people can learn new skills through the Internet [17].

Similar to Easterlin’s paradox, a large part of workers’ job satisfaction stems from comparisons with those around them. By making comparisons with others easy, the Internet reinforces workers’ feelings of superiority or psychological disparity. How is Chinese workers’ job satisfaction affected by Internet use? What exactly are the differences between people with different personal characteristics? We need to explore those questions. We use the China Family Panel Studies (CFPS), a nationally representative and longitudinal survey, which tracks job satisfaction and Internet use of 83,012 samples biennially from 2010 to 2018. Identifying the causal effect of Internet use on job satisfaction has two challenges. The first is omitted variable bias (such as personality traits). While the survey’s panel structure allows us to control for individual fixed effects, there are unobserved and time-varying confounding factors that can potentially bias OLS estimates. Another concern is that the estimates on the effects of Internet use may be biased due to reverse causality. The higher evaluation of job satisfaction may also affect people’s preference of Internet use.

To address these two challenges, we refer to Sabatini and Sarracino instruments, using the urban optical cable line length (km) as an instrumental variable [11]. On the one hand, due to the difference in the use of Internet communication technology, labor force Internet use and Local Area Network (LAN) base station establishment and optical cable transmission speed, which is closely related to the length of the optical cable line; On the other hand, optical cable construction is greatly affected by regional topography, which is generally related to crustal movement and is not affected by job satisfaction. In addition, to address omitted variable bias, we flexibly control for economic variables and include fixed effects at individual and province-year levels. The baseline estimate suggests that compared with those who do not use the Internet, job satisfaction increases by 3.2 percentage points, which is consistent with the growing Internet use in China. Mechanism analysis found that Internet use weakened the negative impact of time efficiency on job satisfaction but strengthened the positive impact of work autonomy on job satisfaction.

Compared to the existing literature, we have made three main contributions. First and foremost, we are the first to examine the mechanism of Internet use on job satisfaction in China. In recent decades, studies have been conducted mainly in Europe, the United States and Southeast Asia. Scholars found that the Internet, as a work tool, can significantly improve job satisfaction [18,19,20], but the impact of the Internet in China is still unknown.

Second, we use the five-period micro-panel data of the CFPS from 2010 to 2018 to analyze the relationship between Internet use and job satisfaction in the urban and rural labor force, which provides important evidence at the micro level for evaluating the economic and social effects of Internet use. The database is widely used to study issues such as Internet use [16,21,22], mental health [23], and income inequality [22,24].

Third, we use the length of urban optical cable lines as an instrumental variable to solve the omitted variable and reverse causality problems and to improve the reliability of the findings. The heterogeneity analysis can help to understand the differences in the impact of the Internet on people with different incomes, education, etc. Finally, the PSM method was used to ensure the robustness of the results.

This paper is organized as follows: Section 2 provides the theoretical analysis and research hypotheses. Section 3 describes the research model and the dataset, followed by the empirical analysis in Section 4. Section 5 proposes two possible mechanisms by which Internet use may affect users’ job satisfaction. Section 6 provides the conclusion.

## 2. Theoretical Analysis and Research Hypotheses

Castellacci and Tveito summarize four channels through which the Internet affects job satisfaction. First, using the Internet may save workers’ work time and increase productivity. Second, through social software, the Internet provides workers with a convenient and inexpensive channel for remote communication. Third, the development of Internet technology has created a large number of new jobs. Fourth, on the Internet, there is a large amount of information and data for workers [18]. We summarize these four channels as two mechanisms that influence job satisfaction: the information acquisition effect and the technology acquisition effect.

The information acquisition effect refers to the fact that the Internet enhances the ability of individuals to access information, communicate information, and information selection in the labor market. First of all, the Internet can showcase and flaunt people’s daily life, which undoubtedly enhances the well-being and job satisfaction of high-income workers. It can also lead to comparison and a desire for material things, which leads to dissatisfaction among low-income employees [15,25]. Second, the knowledge and information that exists on the Internet benefit a segment of the population that lacks information sources. For example, information on agricultural products facilitates farmers to share digital dividends, but it may be false or misleading. Communicating risks can lead to a decrease in well-being [26].

At the same time, the Internet has expanded the scope of information transmission and promoted remote work, thereby enhancing the individual’s sense of well-being. For example, Bloom et al. conducted an experiment with employees of a Chinese Internet company in 2015 and found that job satisfaction increased by 7.22–15.8% for employees who worked from home through the Internet. The authors suggest that this can be explained in terms of both quieter and more convenient work from home [27]. However, there are also studies that suggest that family chores lead to decreased productivity [28]; employees have difficulty distinguishing between family and work boundaries, leading to overwork [29]. Both situations can reduce employee satisfaction.

After the COVID-19 pandemic, many companies tried to encourage employees to work from home via the Internet [30], which provided a natural environment for experimentation. A large number of scholars have pointed out the possible negative impact of remote work on job satisfaction. Vyas and Butakhieo believe that during the home working period caused by the pandemic in Hong Kong, employees were under increased stress and felt exhausted [31]. Gibbs et al. studied a large Asian Internet company and found that work-from-home hours increased significantly during the pandemic, while unit output decreased slightly. They believe there are three explanations: first, working from home is easily interrupted, second, it is more difficult to communicate with colleagues remotely, and third, there is less guidance between leaders and employees [32].

We argue that the above differences are caused by the specific type of work as well as the home environment. Specifically, Bloom et al. studied outcall workers, where the home would be quieter relative to the company. In addition, remote workers who live alone would clearly be less likely to be disturbed than workers with children.

The technology acquisition effect refers to the impact of the Internet on individual skill levels and labor efficiency; the Internet, as the main source of acquiring external knowledge, has improved workers’ skills, so that they can find solutions to tasks without relying on other colleagues [33]. This autonomy is conducive to improving employees’ work motivation and job satisfaction [34].

**Figure 1:**
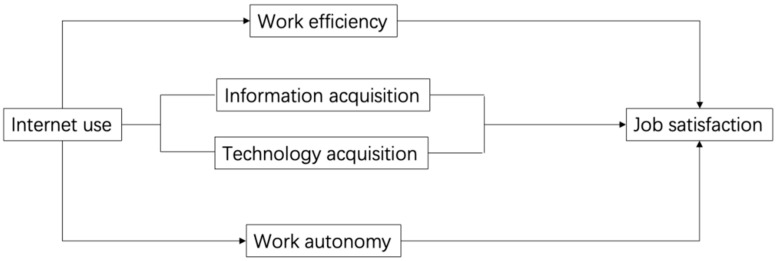
Logical Framework Diagram.

However, there are also studies that have come to different conclusions. Chung-Yan, for example, suggests that the effect of job autonomy on job satisfaction is nonlinear. The positive effect rises with job complexity, but for low difficulty repetitive tasks, job autonomy may lead to lower job satisfaction [35]. Therefore, over-simplicity of work is one of the reasons for negative satisfaction with work autonomy. Based on the above theoretical analysis, we specify a hypothesis concerning Internet use on job satisfaction as follows in Figure 1.

**H1.**  *Internet use can increase job satisfaction*.

As a key factor affecting job satisfaction is work efficiency, most studies show that individual weekly work efficiency positively correlates with job satisfaction, and longer working hours are not conducive to accumulating job satisfaction [36]. Using the European Quality of Life Survey (EQLS), Pichler and Wallace found that workers who were constantly under tight deadlines and low productivity had lower job satisfaction on average [37]; similarly, Lopes et al. found that workers who did not adequately complete their tasks had lower job satisfaction [38]. From the perspective of the information acquisition effect of the Internet, the Internet helps employees perform work tasks in a fast manner through high-speed information dissemination and effective communication; and from the perspective of the technology acquisition effect, the Internet as a powerful office tool can help workers increase productivity and quality of work, and the time saved allows employees to allocate more time to high-reward and interesting tasks, thereby increasing job satisfaction [37,39].

The Internet is a powerful source of access to work information and external knowledge. On the one hand, Dettling Carlson believes that the Internet has expanded the scope of information transmission and promoted the process of telecommuting [40]. When employees perform tasks in a flexible and autonomous way, they are usually more motivated [41]. To a large extent, employees’ job autonomy is defined by their organizational leadership, workplace flexibility, and the way work tasks are organized. The autonomy of work brought by Internet use can help employees choose a more comfortable working environment and working place, expand the limitations of traditional work in space and time, and improve employees’ job satisfaction and work quality [42,43]. On the other hand, the Internet allows employees to carry out their work independently without relying on other colleagues by providing technical help, thus improving job satisfaction. To sum up, using the Internet improves employees’ work autonomy, thus improving job satisfaction.

Based on the above theoretical analysis, we specify a hypothesis concerning the mechanism of Internet use on job satisfaction as follows.

**H2.**  *Internet use increases job satisfaction by increasing work efficiency*.

**H3.**  *Internet use increases job satisfaction by enhancing job autonomy*.

## 3. Dataset and Empirical Strategy

### 3.1. Data Source and Variable Description

We use five waves of the CFPS biennially conducted from 2010 to 2018 to estimate the relationship between Internet use and job satisfaction. The survey reflects the changes in China’s society, economy, population, education, and health by tracking and collecting data at three levels: individual, family and community. We mainly select labor force samples in the age range of 16–64 years and eliminate invalid samples. Among them, Internet usage, job satisfaction, and individual characteristic data come from the adult database, and family characteristics such as family income, family size, family property, and the number of children in the family come from the family database. The adult database and the family database were matched based on the corresponding object code and year, and finally, 83,012 observations were kept.

The explained variable is the job satisfaction of the respondents. Respondents were asked to rate their level of job satisfaction on a scale from 0 to 5, where 0 indicates very dissatisfied and 5 indicates very satisfied.

The explaining variable is Internet use. According to “Do you surf the Internet” in the questionnaire, it includes computer surfing and mobile surfing. The respondent is set to 1 if they do and 0 if they do not.

In addition, the control variables include individual characteristics and family characteristics. Among them, individual characteristics include respondents’ age, age squared term, male, marriage, years of education, health status, popularity of work; family characteristics control family income, family size, family property (family’s other properties other than the current house) and the number of children in the family. The higher the value of health and popularity, the better it is.

Table 1 summarizes the key variables. 36% of people use Internet, their average age is 45 and they are in good health and popularity, and most of them are married. The mean of job satisfaction is 3.5, which means most of the workers are satisfied with their jobs.

### 3.2. Model Settings

Since job satisfaction uses 5-point scales, we use the Ordered Logit model to study the impact of Internet use on job satisfaction of urban and rural laborers. The specific model is as follows:

(1) $$y^{*}{}_{it}=F\left(\beta_{1}Internet_{it}+\beta_{2}X_{it}+\gamma_{it}+\emptyset_{it}+\mu_{it}\right)\;$$

Formula (1), $Internet_{it}$ is whether the urban and rural labor force uses the Internet, the subscript i represents the individual, and t represents the year. $X_{it}$ is a series of control variables and $\gamma_{it}$ and $\emptyset_{it}$ are the fixed effects of individual and county by year. $\mu_{it}$ is the unobservable error term. *F* *(.)* is a nonlinear function whose specific form is as follows:

(2) $$F\left(satisfied_{it}^{*}\right)=\{\begin{array}{c} 1,y_{it}^{*}\leq \mu_{1}\;\;\;\;\;\;\;\;\;\;\\ 2,\mu_{1}<y_{it}^{*}\leq \mu_{2}\\ 3,\mu_{2}<y_{it}^{*}\leq \mu_{3}\\ 4,\mu_{3}<y_{it}^{*}\leq \mu_{4}\\ 5,\mu_{4}<y_{it}^{*}\;\;\;\;\;\;\;\;\;\;\; \end{array}\;\;\;$$

In Formula (2), $\mu_{1}$, $\mu_{2}$... $\mu_{4}$ are the parameters to be estimated, and $y_{it}^{*}$ are the unobservable labor force i’s true job satisfaction in the year, and this variable is a latent variable t.

There can be underlying endogenous issues with Internet usage. On the one hand, there may be a reverse causal relationship between Internet use and job satisfaction. Unobservable omitted variables such as acceptance level; on the other hand, the model may have a self-selection problem when performing regression, and workers may actively choose whether to use the Internet to improve job satisfaction according to their own conditions, preferences, or job nature.

To alleviate the estimation bias caused by the self-selection problem, we also adopt the propensity score matching (PSM) method for estimation. Using the Internet as the treatment group and not using the Internet as the control group, Equation (3) is the probability of the worker sample in the treatment group, and Equation (4) is used to calculate the average effect of using the Internet on job satisfaction.

(3) $$P_{it}\left(X\right)=Pr\left(Internet_{it}=1|X_{it}\right)=L\left[h\left(X_{it}\right)\right]\;\;\;\;\;\;\;\;\;\;\;\;\;\;\;\;\;\;\;\;\;\;\;\;\;\;\;\;\;\;\;\;\;\;$$

(4) $$ATT_{1}=E\left(satisfied_{1}|Internet_{it}=1\right)-E\left(satisfied_{0}|Internet_{it}=1\right)\;\;\;\;$$

## 4. Empirical Results

### 4.1. Baseline Regression Result

Table 2 reports the Ordered Logit estimates of Internet use on job satisfaction. Column (1) of Table 2 studies the regression results of Internet use on job satisfaction of the full sample. The results show that Internet use improves workers’ job satisfaction on an overall level. Column (2) and Column (3) consider the individual fixed effect as well as the county-year fixed effect, with the addition of individual and household control variables. Although the effect of Internet use on job satisfaction decreases from 19.3% to 3.2%, the coefficient is always positive.

The relationship between age and job satisfaction is an “inverted U shape”, indicating that age can improve workers’ job satisfaction, but with the increase in age, its influence on job satisfaction gradually weakens. The higher the education level, the better job satisfaction. Finally, an increase in family size and the number of children in the home can, as expected, negatively affect job satisfaction. This is likely because workers have to take time out of their work to care for children or families, which leads to easy interruptions and reduced efficiency.

In brief, our baseline regression results are consistent with most studies. We decided to conduct heterogeneity analysis for further exploration.

### 4.2. Heterogeneity Analysis

The results in Columns (1) and (2) of Table 3 show that Internet use significantly improves job satisfaction of urban workers, but Internet use has no significant impact on job satisfaction of rural workers. Urban workers are more educated and more likely to engage in multi-tasking and complex jobs that require multiple skills support, and Internet technology as a work tool can enhance urban workers’ performance and job satisfaction [44]. However, these effects are arguably polarizing. Because the rural labor force is mainly manual labor and repetitive tasks, the Internet is not yet its main work tool, and its impact on job satisfaction is limited [45]. This empirical result is basically consistent with the research results of Salanova et al. [46]; that is, different groups are influenced by their educational background and economic status, which leads to large differences in the impact of different groups using the Internet on job satisfaction [47].

There may be differences in the impact of Internet use on job satisfaction among different income groups. Therefore, we divide it into five levels from low to high according to the “local status of personal income” in the CFPS data. The Grouped Ordered Logit regression results are shown in Table 4. The results in Table 4 show that the positive effect of Internet use on job satisfaction is mainly concentrated in the groups with income in the 20–80% quantile range. For both the highest and lowest income groups, there is a negative effect of Internet use on job satisfaction. We argue that the Internet reinforces material desires and income comparisons, which makes the lowest income group dissatisfied with their jobs. Unlike the low-income group, the highest-income group has a low marginal utility of Internet use and more spiritual pursuits, which may lead to insignificant results.

Next, we also examine the effect of Internet use on job satisfaction under different educational backgrounds. The regression results in Table 5 show that with the increase in education level, the impact of Internet use on job satisfaction is also greater. The two are only significantly positively correlated in the group with college education and above and are not significant for primary school and below and middle school education. Maona et al. found that the less educated people have a poorer understanding and absorption of the Internet [48], and they are less likely to see the Internet as a work tool because they may not have sufficient technical literacy or Internet skills. Among the educational groups, the Internet has a limited effect on job satisfaction.

### 4.3. Robustness Checks

Considering reverse causality and missing variable problems, we use robustness checks.

Column (2) of Table 6 is the reduced form regression result, which is a direct regression of job satisfaction to instrumental variables. From the effect of optical cable length on job satisfaction through the Internet, the result is positive and statistically significant, indicating that the longer the optical cable length is, the wider the coverage of communication and information technology, and the greater the positive impact of Internet use on job satisfaction.

Column (3) reports the results of the linear two-stage least squares estimation of the instrumental variables and the results of the F value in the first stage, which shows that Internet use and job satisfaction are significantly positively correlated at the 5% level, and monthly post and telecommunications expenses in the first-stage regression are significantly positively correlated with Internet use. The F value of the first stage is 875.16, greater than the critical value 10, indicating that there is no weak instrumental variable problem. In addition, considering the heteroscedasticity problem at the district and county level, we select the heteroscedasticity robust DWH test with robust standard errors.

The results show that the null hypothesis that there is an endogenous problem in Internet use is rejected at the 1% level. Therefore, these Instrumental variables are valid. In addition, the regression results of urban and rural laborers are basically consistent with those in Table 2 and will not be reported here.

In addition to the endogenous problem caused by omitted variables and reverse causality, the model may also have self-selection problems. Referring to the estimation idea of propensity value matching proposed by Rosenbaum and Rubin, we adopt one-to-one nearest neighbor matching and radius matching based on Formulas (3) and (4) to further identify the causal relationship between the primary item of Internet use skills and the employment quality of migrant workers. As shown in Table 7, in the one-to-one nearest neighbor matching, the average treatment effect of the whole sample is 3.6%, which is significant at the level of 5%; the average treatment effect of the urban labor force is 5.2%, which is significant at the level of 10%; the average treatment effect of rural labor is 1.6%, which is insignificant. In addition, the estimated results of radius matching are basically the same, indicating that the estimated results in we are robust.

**Figure 2:**
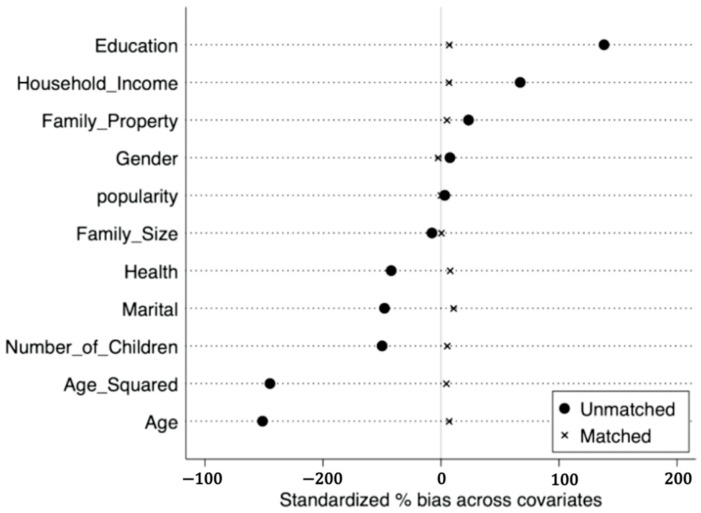
Standardized Deviation Plot for Yes Variables.

**Figure 3:**
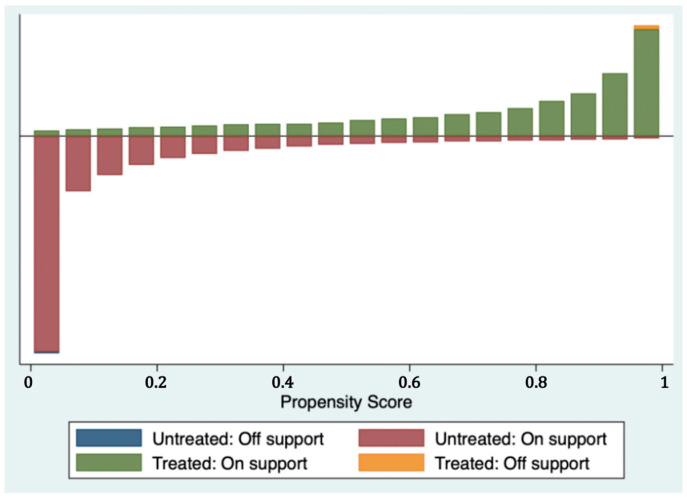
Common Range of Values for Propensity Score Matching.

**Figure 4:**
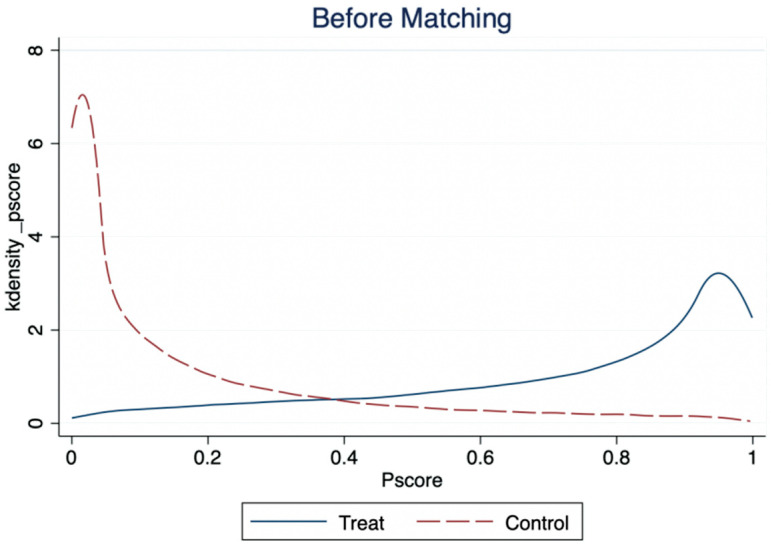

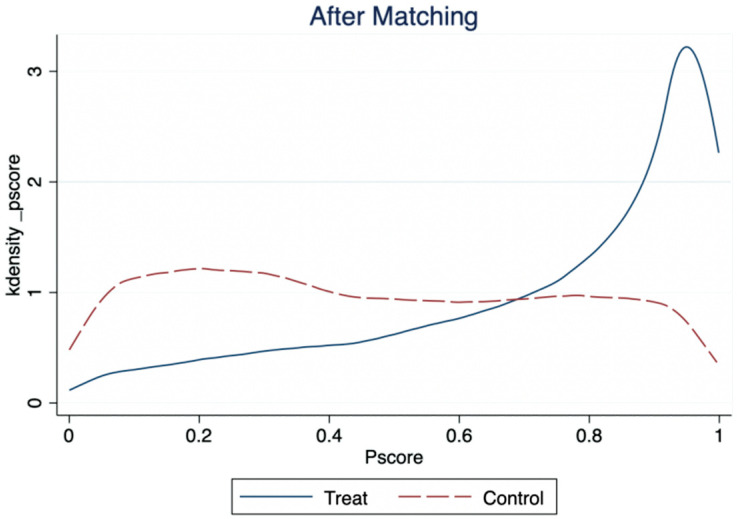
Fit Plot of Propensity Score Before and After Matching.

The standardized deviation diagram of variables before and after matching (Figure 2) shows that the standardized deviations of variables after matching are all less than 10%, indicating that the matching results meet the requirements of balance. Most of the observations are within the common range of values (Figure 3), so only a little sample is lost in the matching, which indicates that the model matches well. In addition, according to the fitting plot of the propensity scores of the treatment and Yes groups before and after matching (Figure 4), the fit between the treatment and Yes groups after matching was better than before matching.

Only keeping a sample of respondents who participated in all five periods, we retain the sample of respondents who participated in all five periods to conduct a regression analysis on the relationship between Internet use and job satisfaction (see Table 8 below). Again, the results are basically the same, and the regression estimation results remain robust.

To comprehensively measure the impact of Internet use on urban and rural labor, we replace the core explanatory variable “Internet use” with respondents’ “attitude to Internet use” for regression estimation. The specific operations are as follows: According to the data on the importance of using the Internet for study, work, social interaction, entertainment, and business activities among respondents in the CFPS adult database, the “Comprehensive Index of Internet Use Importance” is constructed using the entropy method. Table 9 reports the impact of the importance of Internet use on job satisfaction. The regression results are basically consistent with the benchmark regression results, and the results remain robust.

## 5. Mechanism Analysis

### 5.1. Internet Use, Work Efficiency and Job Satisfaction

On average, employees who are constantly under tight deadlines have lower job satisfaction. Based on this, to explore urban and rural labor forces, Internet use will work by saving time to improve work efficiency, and have a positive influence on job satisfaction. We try to use “weekly working time (hours)” to measure the productivity of labor, using the “Internet to work”, which shows that the greater the time pressure, the lower the work efficiency. The corresponding mechanism regression results are reported in Table 10.

The regression results in Column (2) of Table 10 show that labor force Internet use can reduce working hours and improve work efficiency. The results in Column (3) show that labor force Internet use can improve individual job satisfaction by reducing working hours.

### 5.2. Internet Use, Job Autonomy, and Job Satisfaction

We select workplace flexibility that reflects one aspect of work flexibility, with autonomy as 1, and otherwise set to 0. Table 11 reports the corresponding mechanism regression results.

Workplace autonomy can be regarded as one of the important mechanisms affecting Internet use and job satisfaction. Table 11 shows that Internet use significantly improves workplace autonomy, with a 5.4% increase in employee job satisfaction through autonomy. Our study supports the consistent findings of the majority of studies that concluded that the marginal impact of job autonomy ranged from 5% to 17.1% [3,49,50]. We believe the main reasons for the positive impact of job autonomy are innovative workplaces that can increase the diversity and interest of work and make those employed work harder. Meanwhile, workers can also self-coordinate their leisure time, thus increasing their overall job satisfaction.

## 6. Conclusions

We estimate the causal effect of Internet use on job satisfaction using the China Family Panel Studies. We find that Internet use significantly improves job satisfaction by 3.2 percentage points. Our results are robust to different specifications, instrumental strategies, controlling for workers’ educational background and economic status variables, and using alternative measures of both Internet use and job satisfaction. We find evidence that suggests that Internet use induces job satisfaction through strengthening job autonomy and increasing time efficiency.

The findings of this study are: (1) Internet use can significantly improve the job satisfaction of workers, and this effect still holds after robustness analysis. Follow-up mechanism analysis found that Internet use increased job satisfaction through strengthening job autonomy and increasing work efficiency. (2) There are differences in the impact of Internet use on job satisfaction among different income groups. The positive effect of Internet use on job satisfaction is mainly concentrated in the middle-income group, whose individual income is in the 20–80% quantile range. In the low-income class, Internet use will reduce job satisfaction; in addition, we also find that the higher the education level of workers, the more obvious the positive effect of the Internet on job satisfaction; finally, urban workers are more positively impacted by the Internet

The impact of the Internet on job satisfaction is twofold. It will make workers interact less offline but will allow them to have more online interactions. It will both provide a great deal of knowledge for users to learn, but it will also tend to distract workers who become addicted to games, short videos, etc. Despite the many problems with the Internet, our research supports the opinion that the Internet will have more positive effects on job satisfaction.

The Internet should be widely used by companies to improve job satisfaction. The simplest uses are online training, email notifications, etc. In the post-pandemic era, companies can also consider converting some of the work that does not require fixed office space, such as design and telemarketing, into voluntary telecommuting. This will not only reduce the company’s staff density and improve staff motivation, but also reduce some of the site expenses, which is undoubtedly beneficial to the development of the enterprise.

## Figures and Tables

**Table 1:** Descriptive Statistics.

Variable	Mean	Standard Deviation	Minimum	Maximum Value
**Individual Level**				
Job Satisfaction	3.489	0.900	1	5
Internet Use (Yes = 1)	0.364	0.481	0	1
Age	45.004	17.423	9	64
Urban (yes = 1)	0.271	0.444	0	1
Male (Yes = 1)	0.496	0.500	0	1
Marriage (Yes = 1)	0.775	0.418	0	1
Years of Education	7.265	4.880	0	23
State of Health	3.790	1.286	1	5
Popularity at Work	3.934	0.307	1	4
**Family Level**				
Household Income	10.554	1.051	0	15.936
Family Size	4.330	1.970	1	26
Family Property (Yes = 1)	0.178	0.383	0	1
Number of Children in the Family	1.252	1.083	0	10
**Intervening variable**				
Work Efficiency	37.423	25.314	0.1	168
Autonomy	0.345	0.475	0	1

Note: This article is abbreviated at the 1% level, as in the following tables.

**Table 2:** Ordered Logit Estimates of Internet Use and Job Satisfaction.

Dep = Job Satisfaction	Full Sample	Full Sample	Full Sample
	(1)	(2)	(3)
Internet Use	0.193 *** (0.002)	0.194 *** (0.002)	0.032 *** (0.0118)
Age			−0.004 * (0.002)
Age Squared			0.0002 *** (0.0002)
Gender			−0.044 *** (0.008)
Marital Status			0.008 (0.013)
Years of Education			−0.001 (0.001)
State of Health			−0.103 *** (0.004)
Popularity			0.047 ** (0.023)
Household Income			0.040 *** (0.005)
Family Size			−0.013 *** (0.002)
Family Property			0.030 *** (0.011)
Number of Children in the Family			−0.020 *** (0.006)
Individual Fixed Effects	No	Yes	Yes
County-Year Fixed Effect	No	No	Yes
Number of Samples	83,012	83,012	83,012
Pseudo R-Squared	0.116	0.122	0.056

Note: *, **, and *** indicate significance at the 10%, 5% and 1% levels, respectively. In parentheses are the robust standard errors adjusted for White’s heteroskedasticity.

**Table 3:** Urban and Rural Heterogeneity.

Dep = Job Satisfaction	Urban Worker	Rural Worker
	(1)	(2)
Internet Use	0.116 ** (0.056)	0.042 (0.032)
Control Variables	Yes	Yes
Individual Fixed Effects	Yes	Yes
County-Year Fixed Effect	Yes	Yes
Number of Samples	37,399	45,613
Pseudo R-Squared	0.103	0.102

Note: ** indicate significance at the 5% level, respectively. In parentheses are the robust standard errors adjusted for White’s heteroskedasticity.

**Table 4:** Income Status Heterogeneity.

Dep = Job Satisfaction	Income Status Very Low	Income Status Lower	Income Status Medium	Income Status Higher	Income Status Very High
	(1)	(2)	(3)	(4)	(5)
Internet Use	−0.167 ** (0.031)	0.041 ** (0.017)	0.045 ** (0.022)	0.093 ** (0.046)	−0.076 (0.090)
Control Variables	Yes	Yes	Yes	Yes	Yes
Individual Fixed Effects	Yes	Yes	Yes	Yes	Yes
County-Year Fixed Effect	Yes	Yes	Yes	Yes	Yes
Number of Samples	7488	21,912	37,144	10,668	5800
Pseudo R-Squared	0.105	0.102	0.126	0.125	0.072

Note: ** indicate significance at the 5% level, respectively. In parentheses are the robust standard errors adjusted for White’s heteroskedasticity.

**Table 5:** Educational Attainment Heterogeneity.

Dep = Job Satisfaction	Elementary School and Below	Middle School	College and Above
	(1)	(2)	(3)
Internet Use	−0.043 (0.057)	0.018 (0.033)	0.191 * (0.108)
Control Variables	Yes	Yes	Yes
Individual Fixed Effects	Yes	Yes	Yes
County-Year Fixed Effect	Yes	Yes	Yes
Number of Samples	32,176	33,889	16,947
Pseudo R-Squared	0.131	0.124	0.121

Note: * indicate significance at the 10% level, respectively. In parentheses are the robust standard errors adjusted for White’s heteroskedasticity.

**Table 6:** Internet Use and Job Satisfaction: Instrumental Variables.

Dep = Job Satisfaction	OLS	Reduced Form	2SLS Second Stage
	(1)	(2)	(3)
Internet Use	0.032 *** (0.012)		2.611 ** (1.063)
			First Stage: Internet Use
Length of Optical Cable		0.156 *** (0.043)	0.061 *** (0.018)
F Value in the First Stage			875.16
Control Variables	Yes	Yes	Yes
Individual Fixed Effects	Yes	Yes	Yes
County-Year Fixed Effect	Yes	Yes	Yes
Number of Samples	83,012	83,012	83,012
Adj R-Squared	0.056	0.155	0.486

Note: **, and *** indicate significance at the 5% and 1% levels, respectively. In parentheses are the robust standard errors adjusted for White’s heteroskedasticity.

**Table 7:** Internet Use and Job Satisfaction: Propensity Score Matching Method.

Matching Method	Outcome Variable	Treatment Group	Group	ATT	Standard Error	T Value
Full Sample						
One-to-One Nearest Neighbor Matching	Internet Use	3.394	3.358	0.036	0.027	2.31
Radius Match	Internet Use	3.394	3.347	0.047	0.024	2.04
Urban Labor						
One-to-One Nearest Neighbor Matching	Internet Use	3.492	3.440	0.052	0.059	2.19
Radius Match	Internet Use	3.492	3.467	0.025	0.051	2.01
Rural Labor						
One-to-One Nearest Neighbor Matching	Internet Use	3.347	3.346	0.001	0.131	2.05
Radius Match	Internet Use	3.347	3.332	0.016	0.103	2.32

**Table 8:** Robustness Test: Only Retain a Sample of Respondents Who Participated in All Five Periods.

Dep = Job Satisfaction	Full Sample		Urban Labor		Rural Labor	
	O-Logit	2SLS	O-Logit	2SLS	O-Logit	2SLS
	(1)	(2)	(3)	(4)	(5)	(6)
Internet Use	0.088 *** (0.036)	1.155 *** (0.310)	0.104 *** (0.075)	1.125 ** (0.580)	0.068 (0.046)	1.154 (0.462)
Control Variables	Yes	Yes	Yes	Yes	Yes	Yes
Individual Fixed Effects	Yes	Yes	Yes	Yes	Yes	Yes
County-Year Fixed Effect	Yes	Yes	Yes	Yes	Yes	Yes
Number of Samples	58,056	58,056	20,924	20,924	37,132	37,132
R-Squared	0.030	0.453	0.149	0.432	0.103	0.403
Cragg–Donald Wald F		80.781 ***		16.578 ***		46.680 ***

Note: **, and *** indicate significance at the 5% and 1% levels, respectively. In parentheses are the robust standard errors adjusted for White’s heteroskedasticity.

**Table 9:** Robustness Test: The Impact of Internet Use Importance on Job Satisfaction.

Dep = Job Satisfaction	Full Sample		Urban Laborer		Rural Laborer	
	O-Logit	2SLS	O-Logit	2SLS	O-Logit	2SLS
	(1)	(2)	(3)	(4)	(5)	(6)
Importance of Internet Use	0.841 *** (0.049)	4.160 *** (0.142)	0.632 *** (0.092)	0.750 *** (0.277)	0.907 (0.066)	1.406 (0.274)
Control Variables	Yes	Yes	Yes	Yes	Yes	Yes
Individual Fixed Effects	Yes	Yes	Yes	Yes	Yes	Yes
County-Year Fixed Effect	Yes	Yes	Yes	Yes	Yes	Yes
Number of Samples	58,056	58,056	20,924	20,924	37,132	37,132
R-Squared	0.127	0.452	0.134	0.495	0.131	0.464
Cragg–Donald Wald F		17.368 ***		91.880 ***		40.354 ***

Note: *** indicate significance at the 1% level, respectively. In parentheses are the robust standard errors adjusted for White’s heteroskedasticity.

**Table 10:** Internet Use, Work Efficiency and Job satisfaction.

Dep =	Job Satisfaction	Work Efficiency	Job Satisfaction
	O-Logit	OLS	O-Logit
	(1)	(2)	(3)
Internet Use	0.032 *** (0.012)	−0.124 *** (0.032)	0.079 *** (0.034)
Work Efficiency			−0.003 *** (0.001)
Control Variables	Yes	Yes	Yes
Individual Fixed Effects	Yes	Yes	Yes
County-Year Fixed Effect	Yes	Yes	Yes
Number of Samples	83,012	83,012	83,012
R-Squared	0.056	0.124	0.126

Note: *** indicate significance at the 1% level, respectively. In parentheses are the robust standard errors adjusted for White’s heteroskedasticity.

**Table 11:** Internet Use, Job Autonomy and Job Satisfaction.

Dep =	Job Satisfaction	Autonomy	Job Satisfaction
	O-Logit	OLS	O-Logit
	(1)	(2)	(3)
Internet Use	0.032 *** (0.012)	0.006 *** (0.010)	0.161 *** (0.032)
Work Autonomy			0.054 *** (0.041)
Control Variables	Yes	Yes	Yes
Individual Fixed Effects	Yes	Yes	Yes
County-Year Fixed Effect	Yes	Yes	Yes
Number of Samples	83,012	83,012	83,012
R-Squared	0.056	0.107	0.121

Note: *** indicate significance at the 1% level, respectively. In parentheses are the robust standard errors adjusted for White’s heteroskedasticity.

## Data Availability

The data used in this study are provided by the China Household Tracking Survey Database (CFPS), which is open to the public (http://www.isss.pku.edu.cn/cfps/). The datasets generated and analyzed in this study are available from the corresponding author upon reasonable request.
